# Brain-targeted delivery of resveratrol using solid lipid nanoparticles functionalized with apolipoprotein E

**DOI:** 10.1186/s12951-016-0177-x

**Published:** 2016-04-09

**Authors:** Ana Rute Neves, Joana Fontes Queiroz, Salette Reis

**Affiliations:** UCIBIO/REQUIMTE, Department of Chemical Sciences, Faculty of Pharmacy, University of Porto, Rua de Jorge Viterbo Ferreira, 228, 4050-313 Porto, Portugal

**Keywords:** Resveratrol, Solid lipid nanoparticles, Apolipoprotein E, Brain delivery, Blood–brain barrier

## Abstract

**Background:**

The present study takes advantage of the beneficial effects of resveratrol as a neuroprotective compound. Resveratrol-loaded solid lipid nanoparticles were functionalized with apolipoprotein E which can be recognized by the LDL receptors overexpressed on the blood–brain barrier.

**Results:**

Transmission electron microscopy images revealed spherical nanoparticles, dynamic light scattering gave a Z-average lower than 200 nm, and a zeta potential of around −13 mV and very high resveratrol entrapment efficiency (ca. 90 %). In vitro cytotoxic effects were assessed by MTT and LDH assays in hCMEC/D3 cell line and revealed no toxicity up to 50 μM over 4 h of incubation. The permeability through hCMEC/D3 monolayers showed a significant increase (1.8-fold higher) for resveratrol-loaded solid lipid nanoparticles functionalized with apolipoprotein E when compared to non-functionalized ones.

**Conclusions:**

In conclusion, these nanosystems might be a promising strategy for resveratrol delivery into the brain, while protecting it from degradation in the blood stream.

**Graphical abstract:**
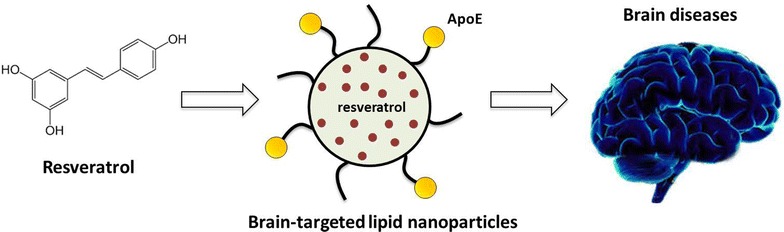
.

**Electronic supplementary material:**

The online version of this article (doi:10.1186/s12951-016-0177-x) contains supplementary material, which is available to authorized users.

## Background

Resveratrol, a stilbene compound catalogued as a polyphenol, has attracted remarkable attention [1]. Among other sources, resveratrol may be found in high concentrations in the grape skins and seeds, mulberries and peanuts [1–3]. Resveratrol can be found as both *cis* and *trans* isomeric forms, being *trans*-isomer the most abundant and biologically active form [2, 4]. Only *trans*-isomer had been consistently linked to health beneficial effects in pharmacological and clinical studies while *cis*-resveratrol had only seen limited success as an antiplatelet agent [5]. *Trans*-resveratrol is an extremely photosensitive compound susceptible to UV-induced isomerization, being rapidly converted to the *cis*-form [1, 4]. This natural compound became popular when the consumption of red wine was linked with the low incidence of cardiovascular diseases, a phenomenon commonly known as the *French Paradox* [6]. Besides cardioprotective effects, resveratrol offers a wide variety of health benefits, such as antioxidant, anti-inflammatory, anti-bacterial, anticarcinogenic activities and neuroprotective effects. Specially, resveratrol seems to be effective in the treatment of neurological disorders, such as Alzheimer’s, Parkinson’s and Huntington’s diseases, brain ischemia, and epilepsy [7–9]. In fact, resveratrol increases antioxidant defense and decreases pro-inflammatory cytokines helping to maintain cerebral homeostasis [1, 10, 11]. However, pharmacokinetic properties of resveratrol are not so favorable, being the in vivo biological effects of resveratrol strongly conditioned due to its poor solubility, chemical instability and rapid metabolism, which reduce its bioavailability [2, 12–14]. As a consequence, the entrance of resveratrol into the brain is limited and this work brings a new strategy to overcome these constrains by developing nanosystems capable of protecting resveratrol from the extensive degradation and metabolism and enhancing the delivery into the brain in an appropriate therapeutic concentration.

Taking into account the high lipophilicity of resveratrol, as well as the high encapsulation efficiency in solid lipid nanoparticles (SLNs) previously reported [3, 7, 15, 16], SLNs were the nanodelivery systems chosen to encapsulate resveratrol and transport it to the brain. These nanoparticles gather the main advantages of the most nanocarriers combined with the high stability of the formulations and their easy preparation avoiding the use of organic solvents [17, 18]. SLNs constitute an attractive colloidal resveratrol carrier system with potential for brain targeting especially when used polysorbate 80 as the surfactant in their preparation [19–24].

Therefore, the aim of this work was the development of resveratrol-loaded SLNs to overcome the problems of low solubility, protecting it from the degradation and, at the same time, targeting it to the brain. For this purpose, resveratrol-loaded SLNs were functionalized with apolipoprotein E (ApoE) using two different strategies previously described in a recent investigation [25]. ApoE-functionalized SLNs may mimic lipoprotein particles that are endocytosed into the blood–brain barrier (BBB) endothelium via low-density lipoprotein (LDL) receptors and transcytosed to the brain [26].

## Results and discussion

Before the functionalization, SLNs with different amounts of resveratrol (0, 2, 5, 10, and 15 mg) were produced and characterized according to their morphology, Z-average, PDI, zeta potential, entrapment efficiency, stability over time and release profile. This information is available in supplementary information. Resveratrol-loaded SLNs were then functionalized with ApoE, as described [25]. The functionalized nanoparticles were also completely characterized and compared with non-functionalized ones. Furthermore, MTT and LDH assays were performed in hCMEC/D3 cell line to evaluate the effect of resveratrol-loaded SLNs in cell viability and cytotoxicity and permeability studies were also conducted in transwell devices using the same cell line.

### Morphology

**Fig. 1:**
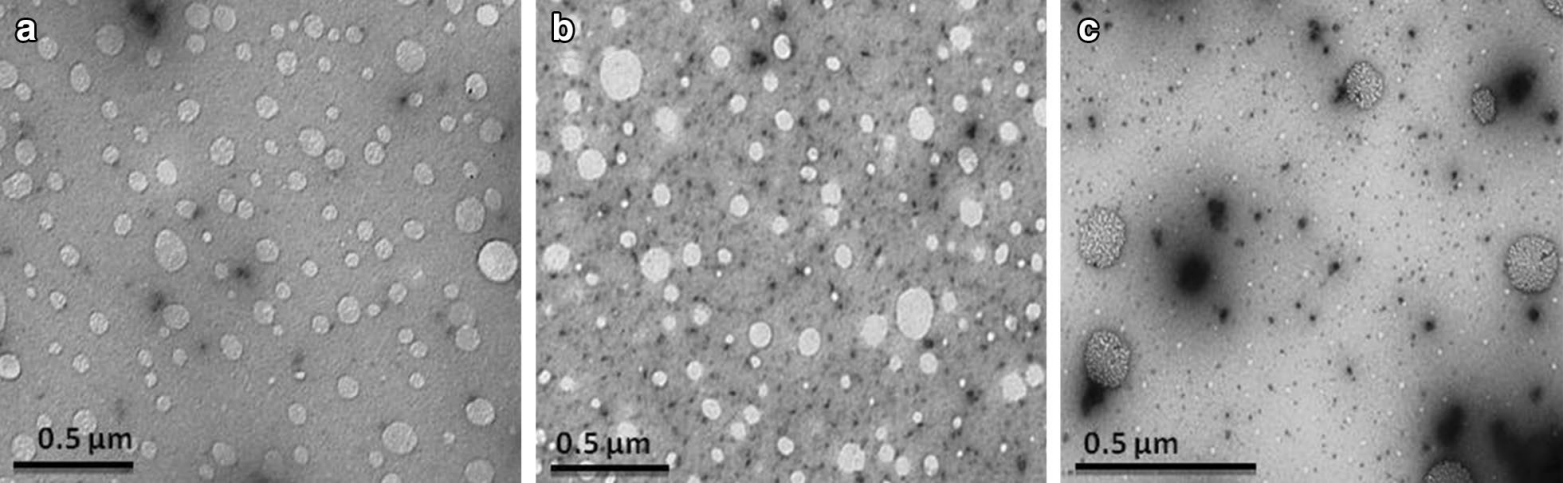
TEM images of resveratrol-loaded SLNs: **a** non-functionalized SLNs; **b** SLN-DSPE-ApoE RSV; **c** SLN-Palmitate-ApoE RSV

The shape and morphology of resveratrol-loaded SLNs were observed by TEM. The images (Fig. 1) reveal that all nanoparticles were almost spherical and uniform in shape. The addiction of ApoE to the nanoparticles surface did not modify its morphology that remained with a spherical shape. The images appear to have an unclear background probably due to the presence of phosphate buffer salt crystals. The mean diameter does not exceed 200 nm for all formulations, although SLN-Palmitate-ApoE seems to have a slightly larger diameter than the others. There was no visible aggregation between them. TEM images also revealed these formulations produced two populations of nanoparticles, the most abundant with a smaller diameter, and other, less pronounced, with a larger size not exceeding 200 nm.

### Particle size and polydispersity

**Fig. 2:**
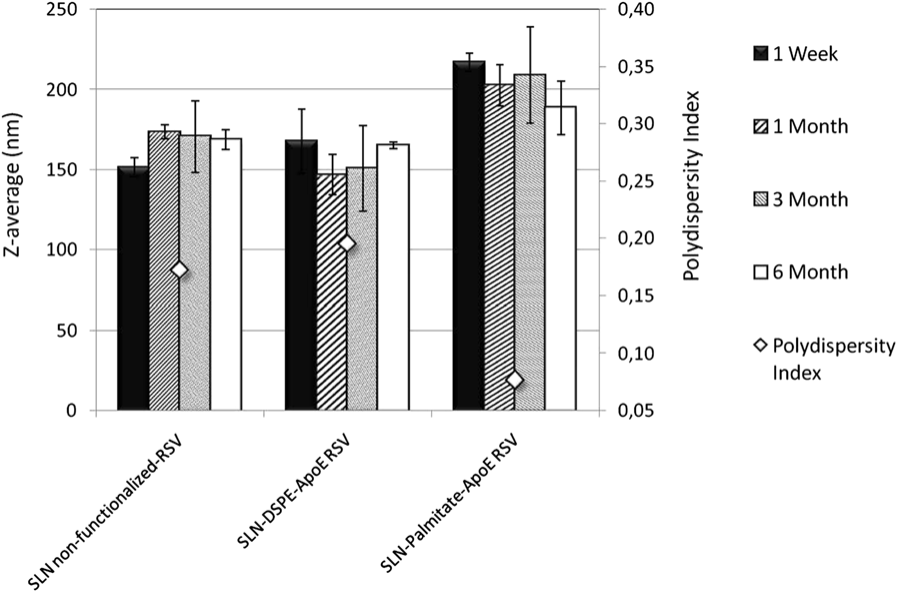
Effect of time of storage on particle size of resveratrol-loaded SLNs non-functionalized and functionalized with ApoE. Z-average after 1 week (), 1 month (), 3 months (), 6 months () and PDI (). All data represent the mean ± SD (n = 3). No statistically significant differences have been found (P > 0.05)

The characterization of resveratrol-loaded SLNs can be seen in Table 1, where Z-average, PDI, zeta potential and EE are presented. Regarding the Z-average, all nanoparticles showed an homogenous size distribution with a mean diameter of about 155 nm and no statistically significant differences between them were observed (P > 0.05), suggesting that resveratrol incorporation does not directly influence the nanoparticles size (Additional file 1: Table S1). The addition of ApoE on the surface of SLNs led to a slight increase in the nanoparticles size but is only statistically significant for the SLN-Palmitate-ApoE (Table 1). These results corroborate the results obtained by TEM where it is noted that the Z-average obtained by DLS is larger than the real size of the nanoparticles (showed by TEM images) once this device measures the hydrodynamic size that includes the hydration sphere surrounding the nanoparticles. As intended, the SLNs produced have an average size less than 200 nm, making them ideal for a long-circulation time and for an increased contact time between the nanoparticles and the BBB, increasing the likelihood to be taken up by the brain [21]. In fact SLNs with a size between 120 and 200 nm are able to escape from reticuloendothelial system (RES) and thus bypass liver and spleen filtration [7]. In relation to PDI, the values obtained were lower than 0.2 for all nanoformulations (Table 1), suggesting that the nanoparticles were in a state of acceptable monodispersity distribution, with low variability and no aggregation. It was also performed a stability study during 6 months to evaluate the stability of the formulations in the shelf conditions. Regarding the size, only small variations occurred over time when increasing the amount of resveratrol, refuting the possibility of aggregation and agglomeration (Additional file 1: Figure S1). For functionalized SLNs, Z-average does not change significantly over time, indicating that there is no aggregation of particles after 6 months of storage (Fig. 2).

**Table 1:** Characterization of resveratrol-loaded SLNs non-functionalized and functionalized with ApoE

Formulation code	Z-average (nm)	PDI	Zeta potential (mV)	Entrapment efficiency (%)
SLN non-funct RSV	151.5 ± 5.8	0.161 ± 0.065	−12.4 ± 4.6	88.7 ± 4.6
SLN-DSPE-ApoE RSV	167.8 ± 19.9	0.195 ± 0.049	−13.05 ± 4.06	98.0 ± 2.5
SLN-Palmitate-ApoE RSV	217.1 ± 5.8*	0.077 ± 0.030	−13.54 ± 1.60	98.9 ± 0.6

All values represent the mean ± standard deviation (n = 3). Results of functionalized SLNs were analyzed and compared with non-functionalized SLNs. (*) denotes statistically significant differences (P < 0.05)

### Zeta potential

**Fig. 3:**
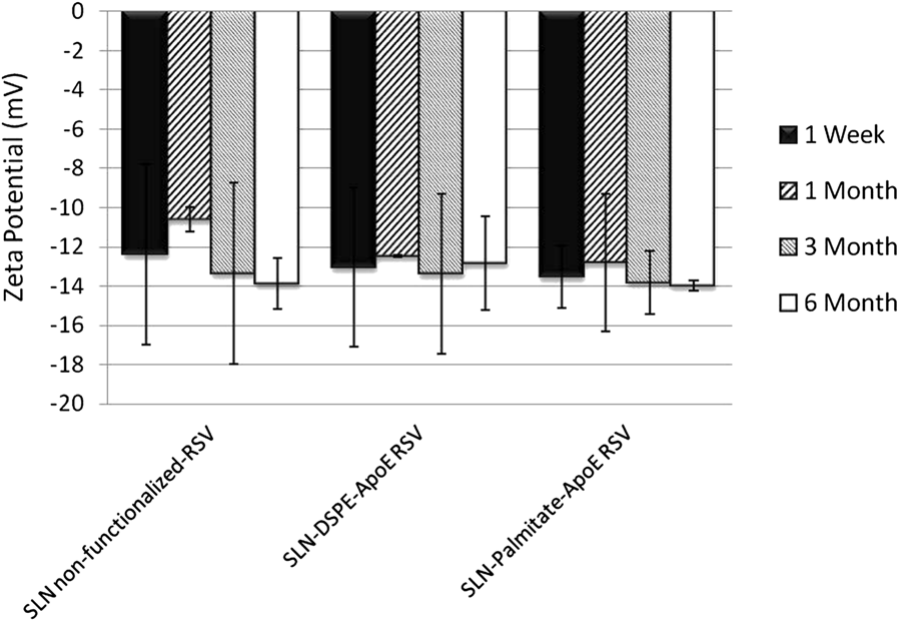
Effect of time of storage on zeta potential of resveratrol-loaded SLNs non-functionalized and functionalized with ApoE. Zeta potential after 1 week (), 1 month (), 3 months () and 6 months (). All data represent the mean ± SD (n = 3). No statistically significant differences have been found (P > 0.05)

Zeta potential is basically the surface charge of the nanoparticles and can be an indicator of the potential stability over time. Nanoparticles have a natural tendency to aggregate, although charged nanoparticles, with high negative or positive zeta potential, will repeal each other overcoming this problem. Therefore, particles can be considered stably dispersed when the absolute value of zeta potential is above 30 mV due to the electrostatic repulsion between the particles, while potentials of 5–15 mV result in limited flocculation in solution [3]. All formulations presented (Additional file 1: Table S1 and Table 1) have a negative average zeta potential between −10 and −15 mV, regardless of resveratrol or ApoE incorporation on lipid nanoparticles (P > 0.05). Therefore, it is essential to perform a stability study to ascertain the formulations stability over time, once the zeta potential is not very high and it may lead to the occurrence of some flocculation and aggregation between the nanoparticles. The zeta potential of the SLNs developed did not significantly change over time (Fig. 3 and Additional file 1: Figure S2). Therefore, despite the initial zeta potential value is not as high as desired, no tendency for zeta potential to change was found during storage conditions, indicating that the SLNs developed will be stable at least for 6 months.

### Resveratrol entrapment efficiency

**Fig. 4:**
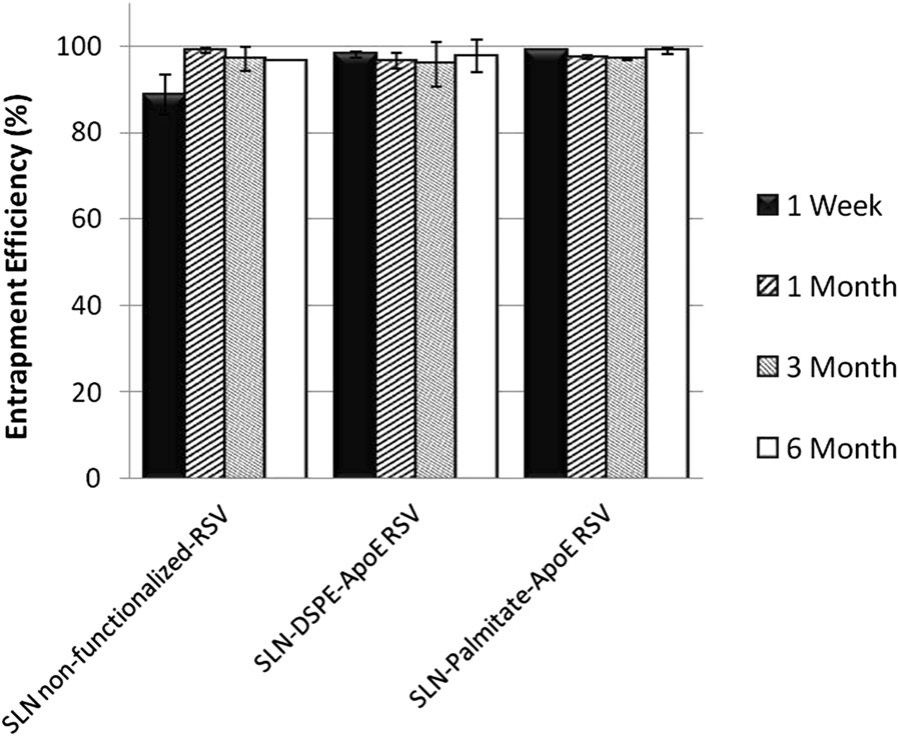
Effect of time of storage on resveratrol entrapment efficiency of non-functionalized SLNs and ApoE-functionalized SLNs. Resveratrol entrapment efficiency after 1 week (), 1 month (), 3 months () and 6 months (). All data represent the mean ± SD (n = 3). No statistically significant differences have been found (P > 0.05)

Resveratrol EE was evaluated to determine the amount of encapsulated resveratrol after SLNs synthesis. Lipid nanoparticles are known to be suitable systems for drug incorporation especially hydrophobic compounds. Therefore, resveratrol lipophilic nature suggests a preferential partition into nanoparticles lipidic matrix. The results for each formulation are shown in Table 1 and EE was found to be satisfactorily high, with an average around 90 %. The values obtained for functionalized SLNs were found to be higher than for non-functionalized ones (Table 1) and, by increasing resveratrol concentration, a decrease in EE is observed as would be expected (Additional file 1: Table S1). By this way, it is possible to state that SLNs are suitable systems for resveratrol incorporation due to the high encapsulation efficiency recorded, well above the values reported in the literature for this type of nanocarriers [27]. The EE was also evaluated over time to ascertain about the physical stability of the lipid nanoparticles. SLNs are characterized by having a highly organized matrix that tends to form perfect crystals over time which can eventually lead to an expulsion of the drug during the storage period. This is the main disadvantage of solid lipid nanoparticles. However, the results of the EE over time indicate no statistically significant decreases in the resveratrol incorporated, after at least 6 months (Fig. 4 and Additional file 1: Figure S3). This suggests that SLNs retain the encapsulated compound over time preventing the possibility of expulsion of the drug, showing once again the great stability of these formulations.

### Release studies

**Fig. 5:**
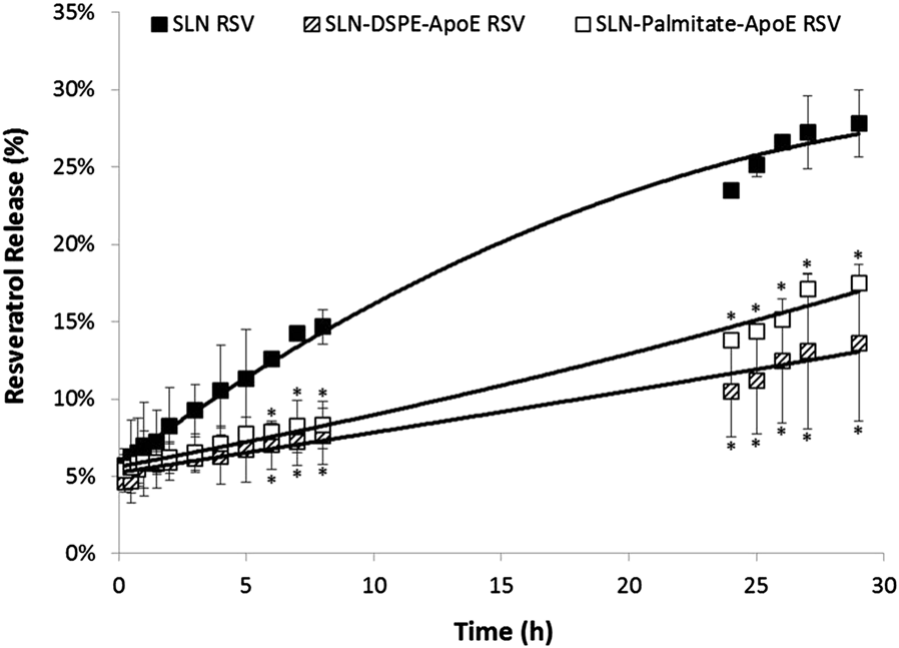
In vitro resveratrol release profiles from non-functionalized SLNs and SLNs functionalized with ApoE performed in SBF, simulating the blood stream conditions, at body temperature (37 °C). All data represent the mean ± SD (n = 3). * denotes statistically significant differences (P < 0.05)

The in vitro release profiles of SLNs can be seen in Fig. 5 and Additional file 1: Figure S4. This study was performed in SBF to simulate the blood stream conditions [28, 29] and represented an important tool for quality control and for predicting the in vivo kinetics, providing an idea of the release profile in the bloodstream. Despite its lipophilic nature, resveratrol has three hydroxyl groups present in its structure, which can lead to a location not only inside the core of the nanoparticles but also at the interface near the shell, favoring an initial burst release [30, 31]. Indeed, about 5–10 % of the total amount of resveratrol was released after only 15 min of incubation with SBF, probably indicating the release of the non-encapsulated compound or the molecules that were merely adsorbed on the surface of the nanoparticles. Statistical analysis showed no significant effect on the release percentages (ca. 20–25 % after 28 h) comparing different initial concentrations of resveratrol (Additional file 1: Figure S4). However, functionalized SLNs reduce the release profile of the compound (ca. 15 % after 28 h) and the initial burst release was not as evident as it was on non-functionalized SLNs. Moreover, it was registered a more controlled release, almost linear, for both types of functionalized SLNs. In fact, the functioning materials, namely the palmitic acid or the phospholipid DSPE, in the SLNs matrix reduce the release of the compound over time, thereby promoting some kind of interaction and stabilization of resveratrol inside the NPs. At the same time, the addition of new groups on the surface of the nanoparticles creates *per se* a steric hindrance avoiding the degradation of the lipid matrix and preventing the premature release of the encapsulated compound. This allows us to conclude that only residual amounts of resveratrol will be released until SLNs to reach their target and that most of the encapsulated compound will be released in a controlled and sustained way.

### MTT and LDH assays

**Fig. 6:**
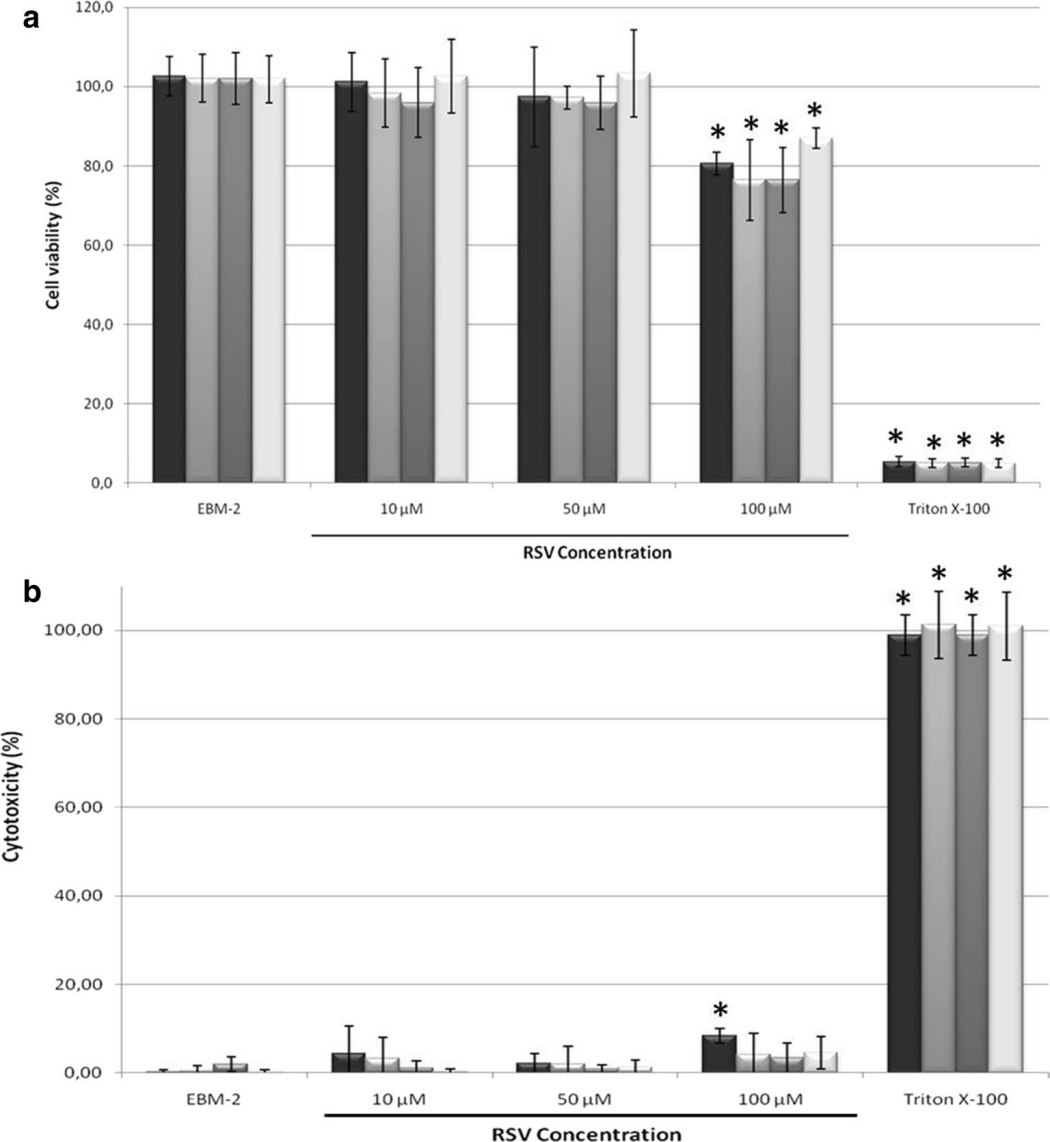
hCMEC/D3 cell viability assessed by MTT assay (**a**) and cytotoxicity assessed by LDH assay (**b**) when exposed for 4 h to non-functionalized SLN RSV (), functionalized SLN-DSPE-ApoE RSV (), functionalized SLN-Palmitate-ApoE RSV () and free resveratrol () at different concentrations of resveratrol (from 10 to 100 μM). All values represent the mean ± standard deviation (n = 3). Results were analyzed and compared with EBM-2 medium which represents the maximum of cell viability and the minimum of cytotoxicity. * denotes statistically significant differences (P < 0.05)

The potential cytotoxicity of resveratrol-loaded SLNs was investigated by MTT and LDH assays on hCMEC/D3 cells (Fig. 6). For 50 μM resveratrol concentration, no statistically significant changes were observed in MTT metabolization or LDH release by cells when compared to the positive control (EBM-2 medium), indicating that up to this concentration SLNs do not affect cell viability or membrane integrity. This result showed that no relevant cytotoxic effects were observed for the concentration used in the permeability studies (50 μM). The viability of cells was only affected by about 20 % at concentrations higher than 100 μM. These evidences are in agreement with the reported effects of this compound, since 50 µM resveratrol increased the oxidative phosphorylation and mitochondrial biogenesis but concentrations higher than 100 μM was described to induce apoptosis and mitochondrial dysfunction, resulting in a significant decrease in cell viability [30].

### Permeability studies

**Fig. 7:**
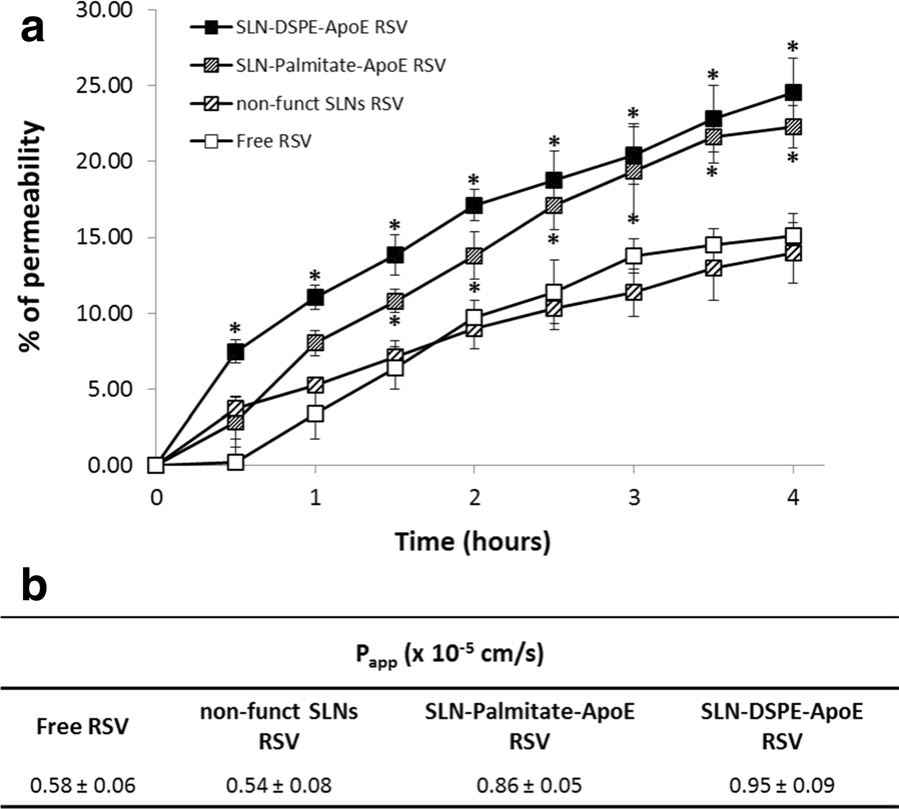
**a** Permeability profile and **b** *P*_*app*_ of free resveratrol (), non-functionalized SLNs RSV (), SLN-Palmitate-ApoE RSV () and SLN-DSPE-ApoE RSV () over 4 h of transport across hCMEC/D3 cell monolayer mimicking BBB permeability conditions. All values represent the mean ± standard deviation (n = 3). Results of resveratrol-loaded SLNs were analyzed and compared to free-resveratrol. * denotes statistically significant differences (P < 0.05)

As described before, hCMEC/D3 cell monolayers exhibit permeability coefficients well correlated with the in vivo permeability data, displaying highly restrictive permeability properties mimicking the in vivo BBB [32, 33]. Hence, hCMEC/D3 cells were cultured in transwell devices and permeability studies were carried out to predict the BBB permeability of resveratrol-loaded SLNs. During the experiments, constant TEER values were obtained around 150 Ω cm^2^, indicating that hCMEC/D3 monolayer maintained its integrity throughout the assay. Moreover, the reported values of *P*_*eff*_ of LY tracer (1.33 × 10^−3^ cm/min) were achieved, once again indicating the proper confluence of cell monolayers. This result reveals that SLNs permeability did not occur by tight junctions opening or monolayer disruption. In Additional file 1: Figure S5, it is possible to see the images recorded for cell growth on transwell membrane, during 7 days after seeding. The permeability profiles and respective* P*_*app*_ values after 4 h of assay are shown in Fig. 7. It is possible to observe that non-functionalized SLNs did not produce a significant change in resveratrol permeability through hCMEC/D3 cell monolayers (ca. 0.55 × 10^−5^ cm/s). However, a significant increase in resveratrol permeability (1.8-fold higher) when encapsulated in ApoE-functionalized SLNs is verified comparing to the non-functionalized ones. It is also possible to notice that there is no significant differences between the maximum permeability values reached after 4 h for each functionalized SLNs, showing that both strategies applied have the same effectiveness in transporting resveratrol through the BBB. By this way, it is possible to state that ApoE conjugation to nanoparticles really enhances resveratrol-loaded SLNs permeability through the BBB which might lead to an increase of resveratrol brain delivery.

## Conclusions

Resveratrol has emerged as a promising natural compound with pleiotropic therapeutic properties and in this work we were particularly interested in its neuroprotective effects. Despite the well-known properties of resveratrol, the ability to produce its neuroprotection effects is largely compromised by its poor pharmacokinetic properties. In fact, resveratrol is chemically unstable, presents low water solubility and is largely metabolized, showing a very low bioavailability [2]. Several approaches have been applied trying to overcome these limitations [13, 34–36], namely the production of nanoparticles capable of protecting it from degradation, thereby increasing topical delivery of resveratrol [37–40] or enhancing oral bioavailability [3, 7, 16]. However, few studies concern the possibility of resveratrol delivery into the brain, being polymeric nanoparticles [41–43] and polymeric nanocapsules containing a lipid-dispersed core [44, 45], the most employed nanosystems. Guo et al. have used transferrin (Tf)-modified polyethyleneglycol-polylactic acid (PEG-PLA) nanoparticles for resveratrol encapsulation, observing a significant decrease of brain tumor volume in C6 glioma-bearing rats [43]. The nanoencapsulation of resveratrol in polysorbate 80-coated poly(lactide) nanoparticles also pointed to a promising nanomedical tool for PD therapy [42]. This coating with polysorbate 80 provided the adsorption of ApoE across blood plasma onto the nanoparticles surface, promoting the recognition by LDL receptors in the brain capillary endothelial cells [42]. In the present study, we were interested in lipid nanoparticles for resveratrol brain delivery, taking advantage of their favorable characteristics, such as their lipophilic nature, their biodegradable and biocompatible composition, and their high physical stability [18]. To the best of our knowledge, only one study in the literature reports the use of such lipid nanoparticles for resveratrol delivery through the BBB, where non-functionalized SLNs significantly increased resveratrol concentration in brain when compared to free resveratrol [46]. In fact, these lipid nanocarriers have shown to be promise as resveratrol delivery systems to enhance the therapy of brain disorders. Therefore, in the present study we have used lipid nanoparticles with covalently attached apolipoprotein E to enhance resveratrol brain delivery, applying two strategies recently developed [25]. This approach can be supported by other studies where apolipoprotein E-enriched nanoparticles can mimic low-density lipoproteins as carriers for brain-specific delivery. For instance, nano-low density lipoproteins have been used as drug delivery vehicles for brain tumors [47]; the brain uptake mechanism of ApoE-coated human serum albumin nanoparticles was also studied [48]; while ApoE-modified liposomal nanoparticles have been reported as promising carriers for gene delivery into the brain [49]. In fact, ApoE Receptor 2 appears to be predominantly expressed in the brain and binds ApoE with high affinity [50–53]. Here, the in vitro cellular studies performed across hCMEC/D3 cells revealed a significant increase of resveratrol permeability (1.8-fold higher) when encapsulated in ApoE-functionalized nanoparticles compared to the non-functionalized ones.

In summary, these nanosystems conferred protection to this compound and increased its permeability through the BBB, which might be a promising strategy for resveratrol delivery into the brain for the treatment of neurological disorders.

## Methods

### Materials

For the nanoparticles synthesis, the solid lipid cetyl palmitate was provided by Gattefossé (Nanterre, France), polysorbate 80 (Tween 80) was supplied by Merck (Darmstadt, Germany) and resveratrol was obtained by Sigma-Aldrich (St Louis, MO, USA). In the preparation of pH 7.4 phosphate buffer solutions, potassium phosphate monobasic was obtained from Sigma-Aldrich and sodium hydroxide from Riedel-de Haën (Seelze, Germany). For the nanoparticles functionalization, sodium deoxycholate, avidin, NHS-palmitate, EDC and ApoE3 were provided by Sigma-Aldrich, DSPE-PEG-NH_2_ was purchased from Avanti Polar Lipids (Alabaster, Alabama, USA) and the biotinylation reagent [EZ-Link Sulfo-NHS (N-hydroxysuccinimido)-Biotin] from Thermo Scientific (Waltham, Massachusetts, USA). For simulated body fluid preparation, dipotassium phosphate was purchased from Sigma-Aldrich; calcium chloride, sodium sulfate and tris base were supplied by Merck; sodium chloride, magnesium chloride and sodium bicarbonate were obtained from Riedel-de Haën; potassium chloride was purchased from Scharlau Chemie S.A. (Barcelona, España); and finally, hydrochloric acid was supplied by Panreac Química (Barcelona, España). For cell culture, phosphate buffered saline (PBS), ascorbic acid, dimethylsulfoxide (DMSO), trypsin, human basic fibroblast growth factor (bFGF), triton X-100 and hydrocortisone were purchased from Sigma-Aldrich. Chemically defined lipid concentrate, HEPES and PenStrep were obtained by Gibco (Carlsbad, CA, USA) and Cultrex Rat Collagen I was purchased by R&D Systems (Minneapolis, USA). EBM-2 endothelial basal medium was obtained by Lonza (Basel, Switzerland) and Fetal Bovine Serum (FBS) “Gold” by PAA.

### Preparation of nanoparticles

Solid lipid nanoparticles were prepared by the high shear homogenization technique followed by sonication, an ultrasound method [3, 16, 18]. The lipid phase containing 500 mg of cetyl palmitate (solid lipid) and 150 mg of polysorbate 80 (surfactant) was melted at 70 °C, in order to reach the lipid’s melting point. Resveratrol (5 mg) was added to the molten lipid and then dispersed in 5 mL phosphate buffer (pH 7.4) at the same temperature, by high-speed stirring in an Ultra-Turrax T25 (Janke and Kunkel IKA-Labortechnik, Staufen, Germany) followed by sonication using a Sonics and Materials Vibra-Cell™ CV18 (Newtown, CT, USA). Particles in the micrometer range were produced by mechanical shearing forces produced by the high-speed stirring in the Ultra-Turrax (120 s at 12,000 rpm), whereas nanoparticles are obtained with the sonication (15 min of 80 % intensity).

### Functionalization of nanoparticles with apolipoprotein E

The functionalization of SLNs with ApoE took advantage of the strongest known non-covalent interaction between avidin and biotin. The process started by the addition of the functionally active avidin onto the surface of SLN. This addition of avidin was achieved by two different strategies: using DSPE-PEG-Avidin or Palmitate-Avidin, as described before [25]. Briefly, in the first strategy, SLNs were prepared incorporating 10 mg of DSPE-PEG-NH_2_ in the lipid phase. The amino terminal group exposed on the SLNs surface was subsequently conjugated to a carboxyl group of avidin (5 mg/mL) forming a peptide bond. In the second strategy, a 15-fold molar excess of NHS-palmitate was added to an avidin solution (5 mg/mL) and incubated at 37 °C for 3 h. SLNs were then prepared as previously described incorporating Palmitate-Avidin (2 mL) in the lipid phase. The binding of ApoE to the SLNs surface was carried out by spontaneous interaction between the previously biotinylated ApoE and the covalently attached avidin on the SLNs surface (30 min, RT), resulting in two different ApoE-functionalized SLNs: SLN-DSPE-ApoE and SLN-Palmitate-ApoE. The resulting functionalized formulations were then dialysed (10 K MWCO) against PBS (37 °C) to remove the excess subtracts and by-products. The confirmation of ApoE functionalization was achieved by infra-red spectra analysis on the lyophilized samples using Fourier Transform Infrared Spectroscopy and by fluorimetric assays using a fluorescent probe (biotinylated fluorescein) to quantify the available biotin-binding sites on the SLNs surface, as described elsewhere [25].

### Morphology determination

In an attempt to determine and evaluate the morphology of the SLNs developed, samples were analyzed by transmission electronic microscopy (TEM). The samples were mounted on 300 mesh form var copper grids, stained with uranyl acetate, and were examined using a Jeol JEM 1400 transmission electron microscope (Tokyo). Images were digitally recorded using a Gatan SC 1000 ORIUS CCD camera (Warrendale, PA, USA), and photomontages were performed using Adobe Photoshop CS software (Adobe Systems, San Jose, CA).

### Particle size and zeta potential measurements

Particle size analysis was conducted by dynamic light scattering (DLS), using a particle size analyzer (Brookhaven Instruments, Holtsville, NY, USA). Mean hydrodynamic diameter (Z-average), the size distribution and polydispersity index (PDI) of the nanoparticles in suspension were assessed by this technique. Zeta potential was determined by electrophoretic light scattering (ELS) using a ZetaPALS zeta potential analyzer (Brookhaven Instruments, Holtsville, NY, USA). Prior to the measurements, all samples were diluted (1:200) in PBS (pH 7.4) to yield a suitable scattering intensity. The average count rate was always between 100 and 500 kcps, showing that the dilution applied to the formulations was appropriate. The Z-average, PDI and zeta potential were obtained by calculating the average of ten runs of three independent batches of SLNs.

### Resveratrol entrapment efficiency (EE)

To determine the EE of resveratrol, an indirect method was used to quantity the amount of free compound which still remains in the aqueous phase. SLNs samples were diluted in phosphate buffer (1:200) and filtrated assisted by centrifugation for 10 min at 3300 g in Amicon^®^ Ultra-4 Centrifugal Filter Devices (Millipore, Billerica, MA, USA) using a Jouan BR4i multifunction centrifuge with a KeyWrite-D™ interface (Thermo Electron, Waltham, MA, USA) with a fixed 23°-angle rotor. Nanoparticles were then retained in the filter while the total amount of non-encapsulated resveratrol were present in the supernatant and quantified using a V-660 spectrophotometer (Jasco, Easton, MD, USA) at 305 nm. The EE was then calculated:

$$EE = \frac{{{\text{Total amount of resveratrol }}-{\text{Nonencapsulated resveratrol}}}}{\text{Total amount of resveratrol}} \times 100$$

### In vitro resveratrol release studies

The in vitro resveratrol release studies were performed using Float-A-Lyzer G2 dialysis devices (Spectrum^®^ Laboratories, Inc) with a biotech grade cellulose ester dialysis membrane (with a nominal molecular weight cut off of 3.5–5 kD). Our aim was to mimic the bloodstream conditions to simulate the release of resveratrol from nanoparticles during its traffic until it reaches their target (BBB). For this purpose, it was used a simulated body fluid (SBF) developed by Kokubo and his team that has inorganic ion concentrations nearly equal to those of human blood plasma and is buffered at pH 7.4 [28, 29]. Diluted samples (1:12, v/v) were incubated for 28 h in SBF at body temperature (37 °C) while being stirred at 100 rpm. At regular intervals, aliquots were collected and replaced with the same volume of fresh medium of SBF to maintain the sink conditions. The amount of resveratrol released was quantified using a Synergy™ HT Multi-mode Microplate Reader (BioTek Instruments Inc, Winooski, VT, USA). The release studies were conducted in quadruplicate and the cumulative percentage of the released compound was determined by calculating the average of all the experiments.

### Cell studies

Immortalized human cerebral microvascular endothelial cells (hCMEC/D3) were obtained under license from Institut National de la Santé et de la Recherche Médicale (INSERM, Paris, France). This cell line shows a similar morphology to primary cultures of brain endothelial cells, representing one such model of the human BBB that can be easily grown and is very useful to cellular and molecular studies, being an important research tool for the characterization of the interactions between drugs or formulations and brain endothelial cells [32, 33]. hCMEC/D3 cells between passage number 26 and 34 were used in all studies, since they maintain stable growth and endothelial marker characteristics, at least until passage 35 [33]. Cells were seeded in a concentration of 2.5 × 10^4^ cells/cm^2^ and grown at 37 °C, in an atmosphere of 5 % CO_2_ in EBM-2 medium supplemented with 1 % Pen-Strep, 2 % FBS gold, 1 % chemically defined lipid concentrate, 5 μg/ml ascorbic acid, 1.4 μM hydrocortisone, 10 mM HEPES and 1 ng/ml bFGF. 0.1 mg/mL rat collagen type I was used to pre-coat all flasks, 1 h at 37 °C. Cell culture medium was changed every 2–3 days. Cell pictures were recorded using an inverted microscope (Motic AE2000 TRI coupled with a camera Moticam 5 MP; Spain).

### MTT and LDH assays

To access the cell viability after SLNs exposition, it was performed the MTT assay, since the level of MTT cleavage in formazan by cells is directly proportional to the number of live and viable (metabolic active) cells [54]. LDH assays were also conducted in order to evaluate the cell death levels and more specifically the potential of SLNs in causing damage in cell membrane. This technique allows the lactate dehydrogenase (LDH) quantification, an enzyme that is released from cells to the surrounding cell culture supernatant when there is damage or rupture of the cell cytoplasmic membranes. Cells were seeded in 96-well plates (10^4^ cells per well) pre-coated with type I collagen. After 20 h of incubation at 37 °C and 5 % CO_2_, different concentrations of free resveratrol and resveratrol-loaded SLNs (functionalized and non-functionalized) were incubated with the cells for 4 h. Positive and negative controls were also used to ensure the reliability of this technique. EBM-2 medium represents the maximum of cell viability and the minimum of cytotoxicity, while triton-X 100 represents the minimum of cell viability and the maximum of cytotoxicity. After the exposition time, the medium of each well was separated from the cells and stored for further use in LDH assay and cells were treated with 0.5 mg/ml of MTT for 4 h, at 37 °C and 5 % CO_2_. DMSO was added to each well to dissolve MTT formazan and plates were incubated for more 15 min at 37 °C, in the dark. The medium resulting from the incubation of samples with cells were collected and centrifuged (250 g for 10 min, at room temperature) and the supernatant separated from the deposited cells in each well. With this centrifugation, it is removed any wastes and cellular debris and also SLNs. The LDH release into culture supernatants was detected by adding catalyst and dye solutions of LDH cytotoxicity detection kit (Takara Bio Inc, Shiga, Japan), after an incubation of 20 min at room temperature in the dark. Absorbance was read at 490 and 690 nm using a Synergy™ HT Multi-mode Microplate Reader (BioTek Instruments Inc, Winooski, VT, USA). Cell viability and cytotoxicity were expressed as a percentage derived from the ratio of the absorbance of treated cells compared to the control obtained by incubating cells only with culture medium (EBM-2) at the same conditions.

### Permeability studies

For permeability experiments, hCMEC/D3 cells were seeded on transwell filters pre-coated with type I collagen (polyester 6 well, pore size 0.4 µm and a diameter of 4.67 cm^2^) in a density of 2 × 10^5^ cells per insert. Cell medium was changed 3 days after seeding and the permeability assay was performed 7 days after seeding. In order to ensure the integrity of hCMEC/D3 monolayer, the transendothelial electrical resistance (TEER) was monitored with an epithelial voltohmmeter (EVOM) from World Precision Instruments (Sarasota, FL, USA). In addition, the quality of the monolayers was tested by measuring the effective permeability (*P*_*eff*_) of lucifer yellow (LY), a highly hydrophilic test compound, comparing the values obtained with the previously reported ones [55]. The *P*_*eff*_ of LY was found to be 1.32 ± 0.07 × 10^−3^ cm/min (when 20 µM of LY were applied on the apical donor compartment) which is consistent with the 1.33 × 10^−3^ cm/min found in literature, meaning the suitable monolayer confluence for the assays performed with resveratrol-loaded SLNs [55, 56].

For the permeability studies, free resveratrol (50 μM) and resveratrol-loaded SLNs (functionalized and non-functionalized with ApoE) were incubated in the apical donor compartment for 4 h at 37 °C and 5 % CO_2_ atmosphere. The concentration of resveratrol in the receptor compartment was quantified every 30 min during 4 h, by fluorescence analysis (330/374 nm) and the apparent permeability coefficients (*P*_*app*_) of SLNs were calculated by the equation $P_{app} \left( {{{cm} \mathord{\left/ {\vphantom {{cm} s}} \right. \kern-0pt} s}} \right) = \frac{Q}{A . C . t}$, where *Q* represents the total amount of permeated resveratrol (μg) in each time point, *A* is the surface area of the membrane filter (cm^2^), *C* is the initial resveratrol concentration (μg/cm^3^) and *t* is the experiment time (s).

### Statistical analysis

The statistical analysis was performed using SPSS software (v 22.0; IBM, Armonk, NY, USA). The values obtained represent the average of at least three experiments and data were expressed as mean ± SD. Data were analyzed using one-way analysis of variance (one-way ANOVA), followed by Bonferroni and Tukey post hoc tests. A P value of 0.05 was considered statistically significant.

## Additional file

10.1186/s12951-016-0177-x Characterization of Resveratrol-loaded SLNs: SLN placebo and SLN-Resveratrol (2, 5, 10 and 15 mg of resveratrol). **Figure S1**. Effect of time of storage on particle size of RSV loaded SLNs with different amounts of RSV. **Figure S2**. Effect of time of storage on zeta potential of RSV loaded SLNs with different amounts of RSV. **Figure S3**. Effect of time of storage on entrapment efficiency of RSV loaded SLNs with different amounts ofRSV. **Figure S4**. In vitro RSV release profiles from RSV loaded SLNs with different amounts of RSV performed in SBF, simulating the blood stream conditions, at body temperature (37 ºC). **Figure S5**. Images of hCMEC/D3 cells on transwell devices during the 7 days of growing.
