# Supplementary material for: Brain-targeted delivery of resveratrol using solid lipid nanoparticles functionalized with apolipoprotein E
Source: J Nanobiotechnology. 2016 Apr 9;14:27. doi: 10.1186/s12951-016-0177-x (PMC4826547; doi:10.1186/s12951-016-0177-x)
Supplement: Supplementary file 1 — 10.1186/s12951-016-0177-x Characterization of Resveratrol-loaded SLNs: SLN placebo and SLN-Resveratrol (2, 5, 10 and 15 mg of resveratrol). Figure S1. Effect of time of storage on particle size of RSV loaded SLNs with different amounts of RSV. Figure S2. Effect of time of storage on zeta potential of RSV loaded SLNs with different amounts of RSV. Figure S3. Effect of time of storage on entrapment efficiency of RSV loaded SLNs with different amounts ofRSV. Figure S4. In vitro RSV release profiles from RSV loaded SLNs with different amounts of RSV performed in SBF, simulating the blood stream conditions, at body temperature (37 ºC). Figure S5. Images of hCMEC/D3 cells on transwell devices during the 7 days of growing. [file 12951_2016_177_MOESM1_ESM.docx]

**Supplementary Data**

**Brain-targeted delivery of resveratrol using solid lipid nanoparticles functionalized with apolipoprotein E**

**Ana Rute Neves^1a^, Joana Fontes Queiroz^1a^, and Salette Reis^1^***

^1^ REQUI*M*TE, Department of Chemical Sciences, Faculty of Pharmacy, University of Porto, Rua de Jorge Viterbo Ferreira, 228, 4050-313 Porto, Portugal.

^a^ These authors have contributed equally to this work.

*** Corresponding author:**

Salette Reis

Department of Chemical Sciences, Faculty of Pharmacy of University of Porto

Rua de Jorge Viterbo Ferreira, 228, 4050-313 Porto, Portugal

TEL: (+351)220428672

FAX: (+351)226093390

E-mail: shreis@ff.up.pt

| Table S1. Characterization of RESVERATROL-loaded SLNs: SLN placebo and SLN –RESVERATROL (2,5,10 and 15 mg of RESVERATROL). | | | | |
| --- | --- | --- | --- | --- |
| Formulation Code | Z-average (nm) | PDI | Zeta Potential (mV) | Entrapment Efficiency (%) |
| SLN Placebo | 151.2 ± 12.0 | 0.191 ± 0,060 | -12.5 ± 2.8 | **-** |
| SLN- RESVERATROL 2mg | 157.4 ± 14.9 | 0.142 ± 0.032 | -13.0 ± 0.9 | 95.6 ± 4.0 |
| SLN- RESVERATROL 5mg | 151.5 ± 5.8 | 0.161 ± 0.065 | -12.4 ± 4.6 | 88.7 ± 4.6 |
| SLN- RESVERATROL 10 mg | 162.1 ± 6.8 | 0.124 ± 0.061 | -12.1 ± 1.3 | 83.2 ± 1.7 |
| SLN- RESVERATROL 15 mg | 174.0 ± 5.0 | 0.108 ± 0.040 | -12.4 ± 0.5 | 67.5 ± 10.0 |

**Note:** All values represent the mean ± standard deviation (n = 3). Results of SLN Placebo were analyzed and compared with RESVERATROL- SLNs. No statistically significant differences have been found (P > 0.05).


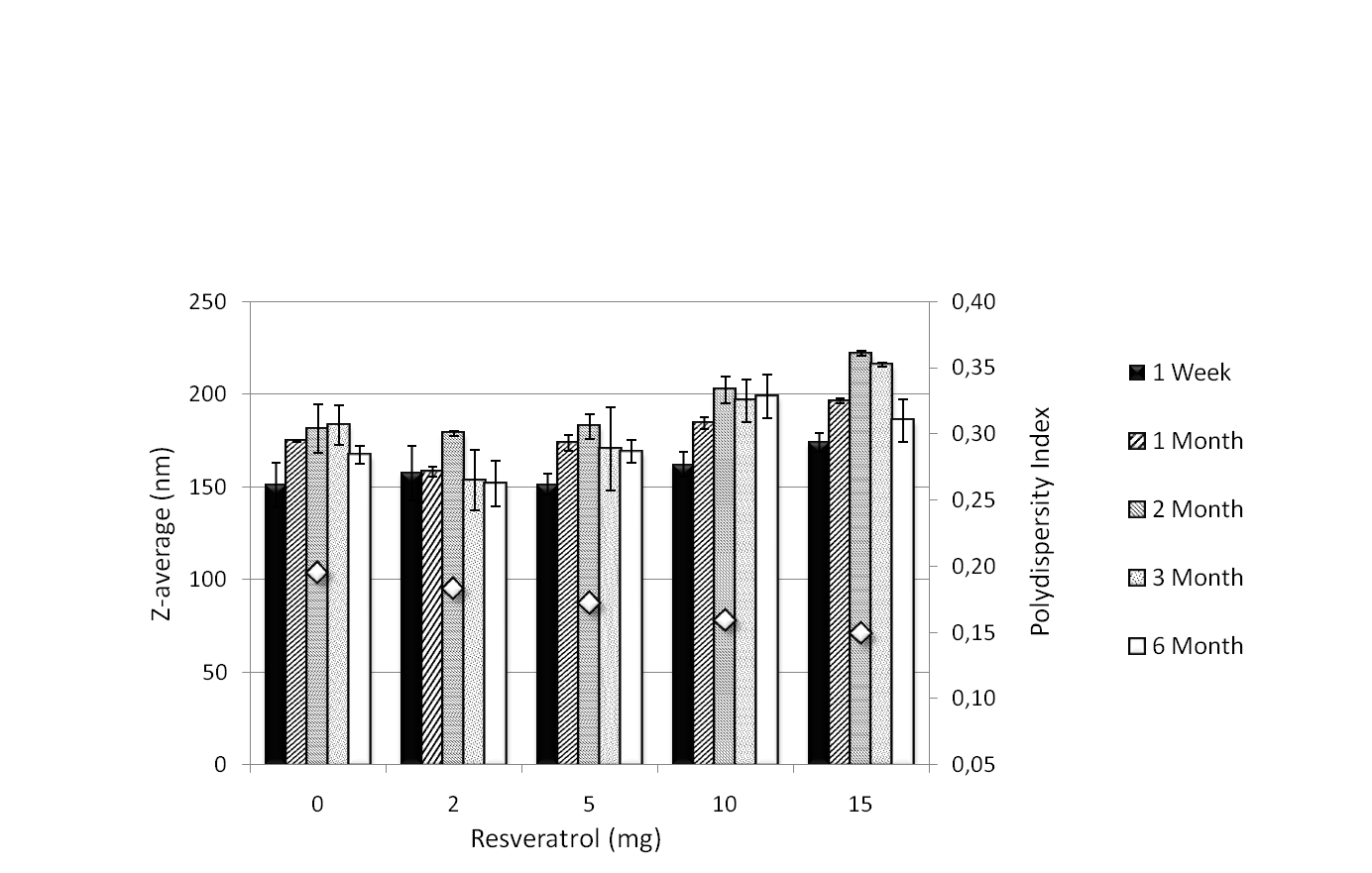


*

*

*

*

*

*

*

*

*

*

**Figure S1 –** Effect of time of storage (at 10°C) on particle size of RSV loaded SLNs with different amounts of RSV. **Note:** Z-average after 1 week ( ), 1 month ( ), 2 months ( ), 3 months ( ), 6 months ( ) and PDI (
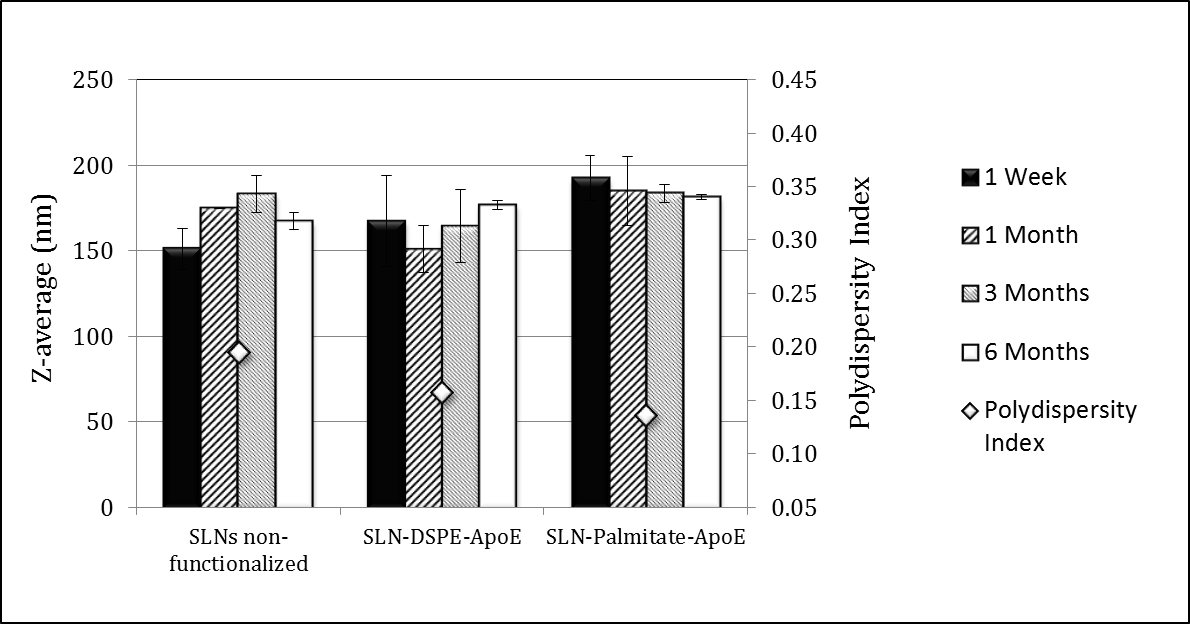
). All data represent the mean ± SD (n=3). (*) denotes statistically significant differences (P < 0.05).


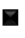

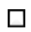

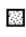

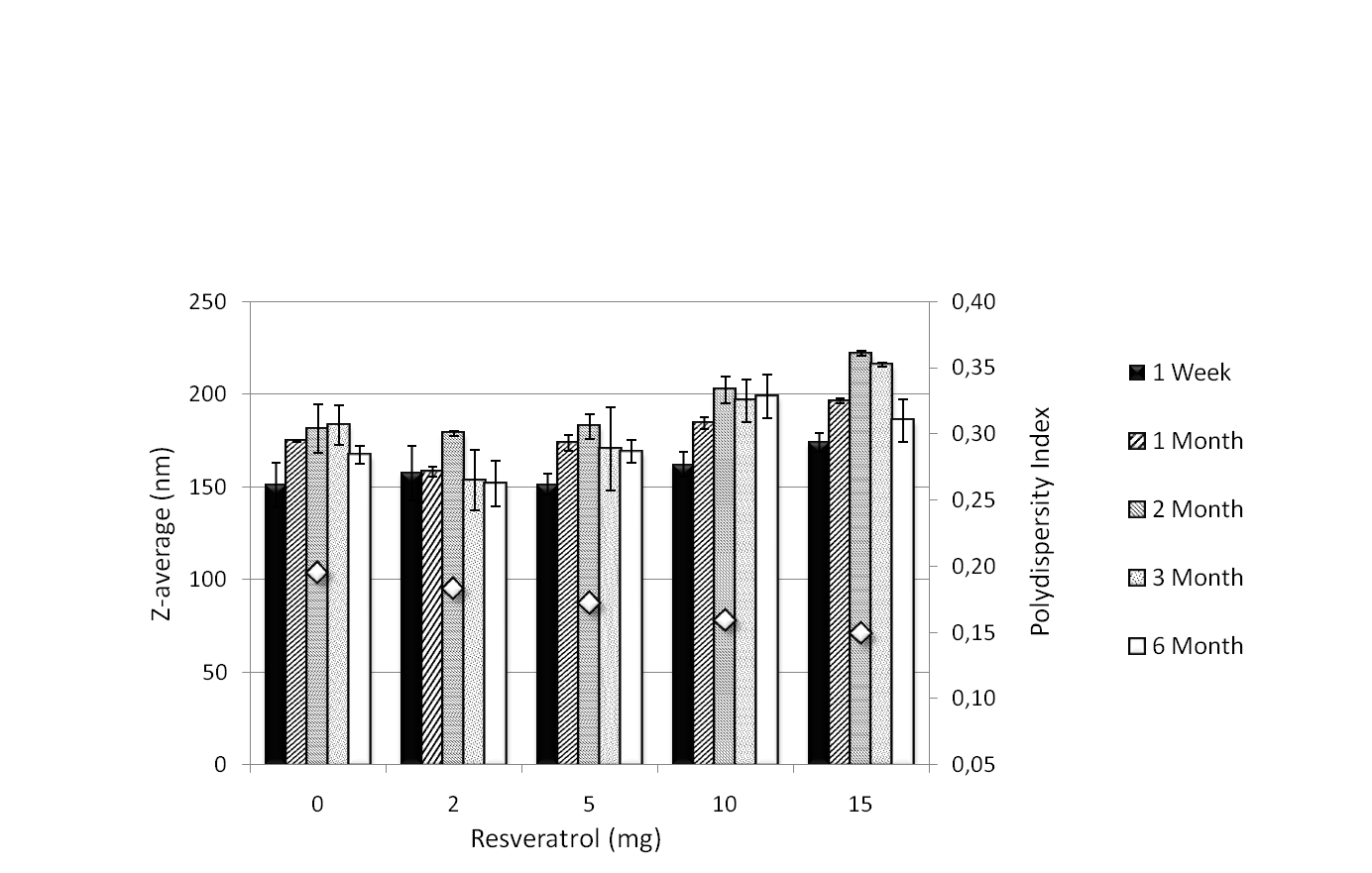

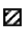


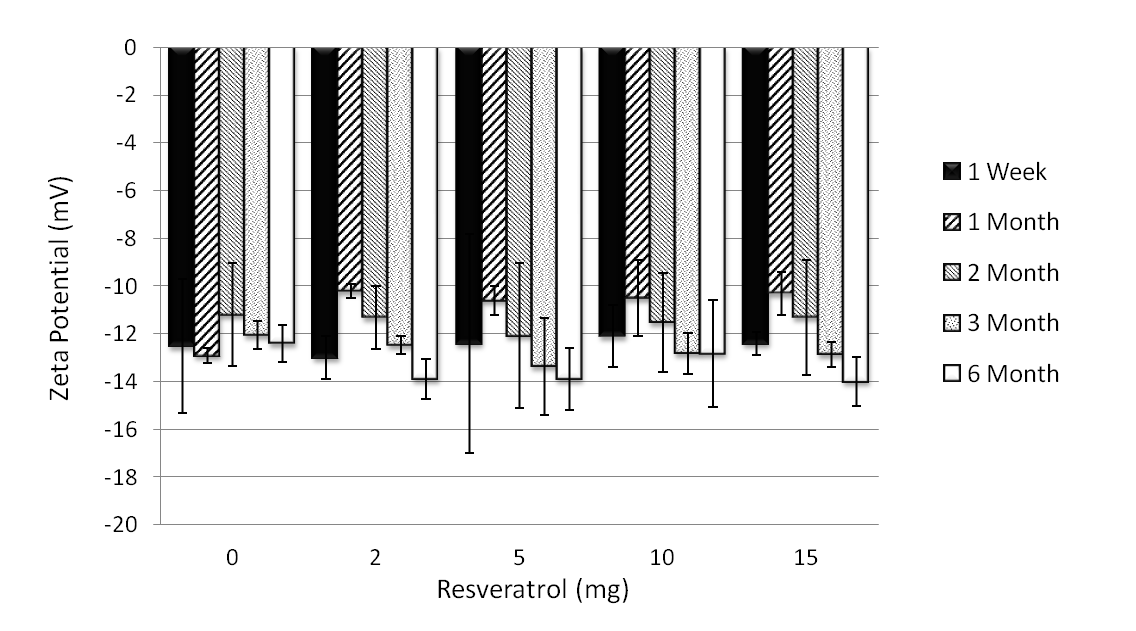


**Figure S2 –** Effect of time of storage (at 10°C) on zeta potential of RSV loaded SLNs with different amounts of RSV. **Note:** Zeta potential after 1 week ( ), 1 month ( ), 2 months ( ), 3 months ( ) and 6 months ( ). All data represent the mean ± SD (n=3). No statistically significant differences have been found (P > 0.05).


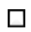

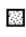

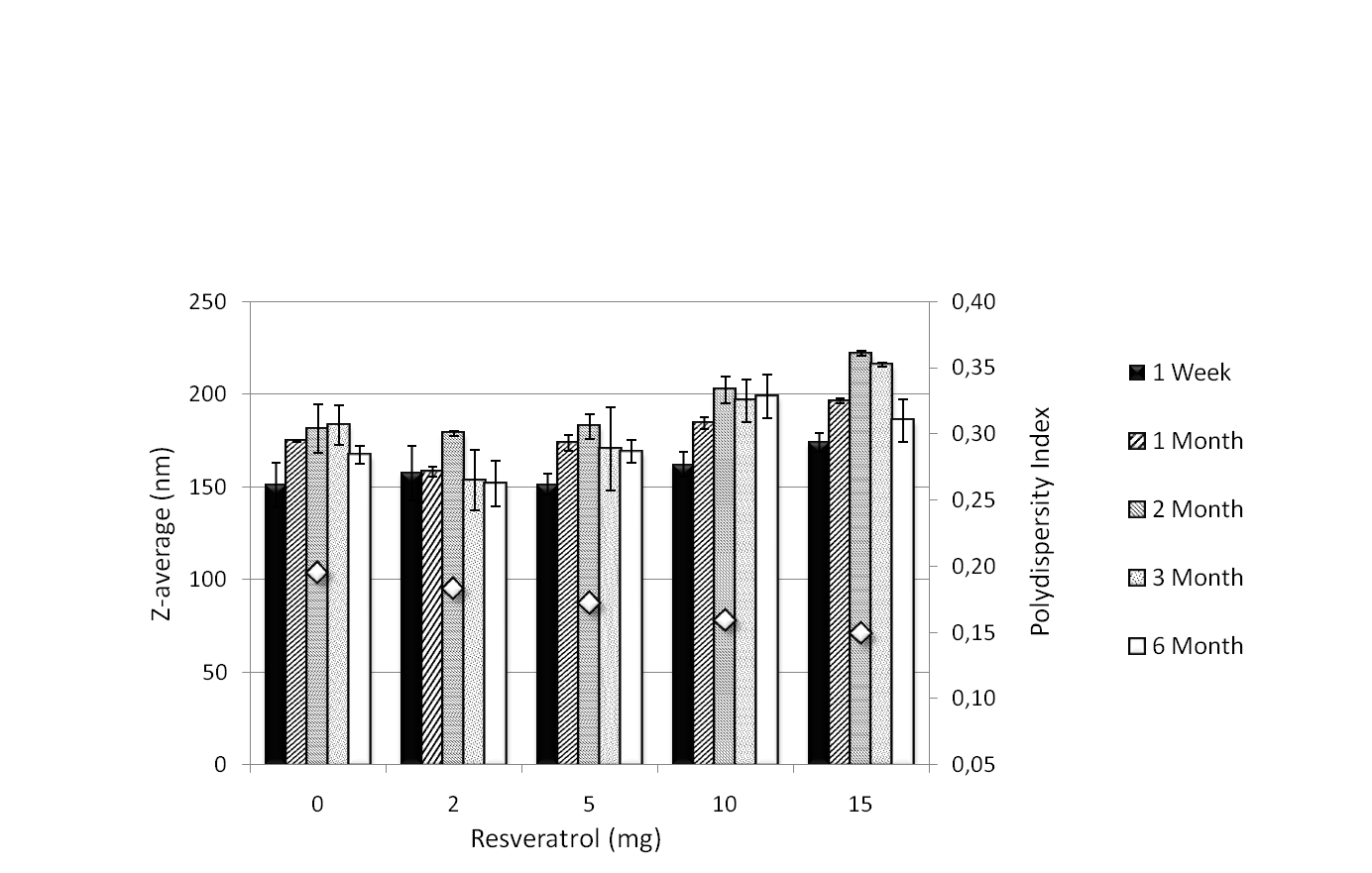

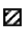

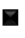


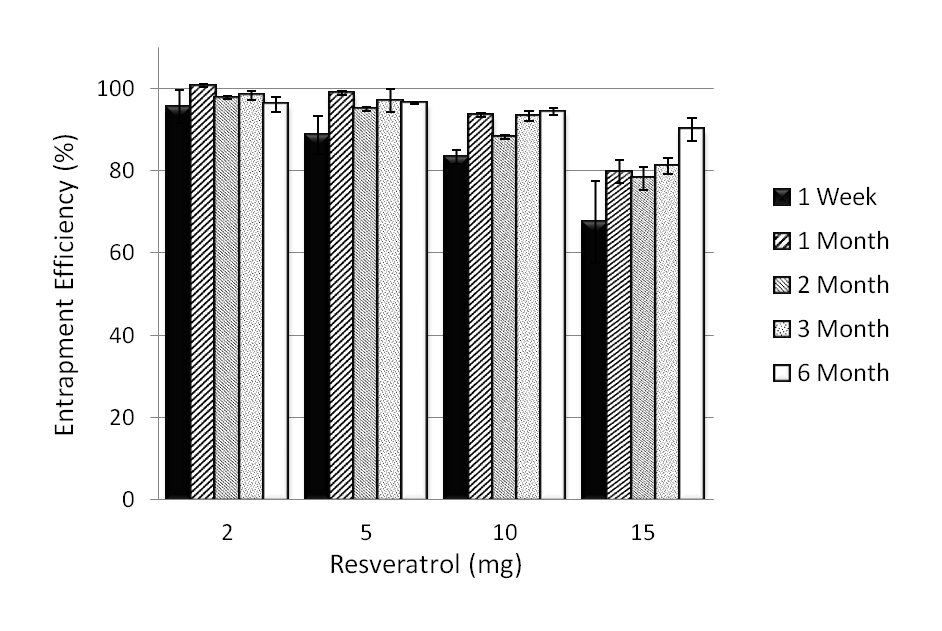


*

*

*

*

**Figure S3 –** Effect of time of storage (at 10°C) on entrapment efficiency of RSV loaded SLNs with different amounts of RSV. **Note:** RSV entrapment efficiency after 1 week ( ), 1 month ( ), 2 months ( ), 3 months ( ) and 6 months ( ). All data represent the mean ± SD (n=3). (*) denotes statistically significant differences (P < 0.05).


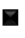

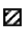

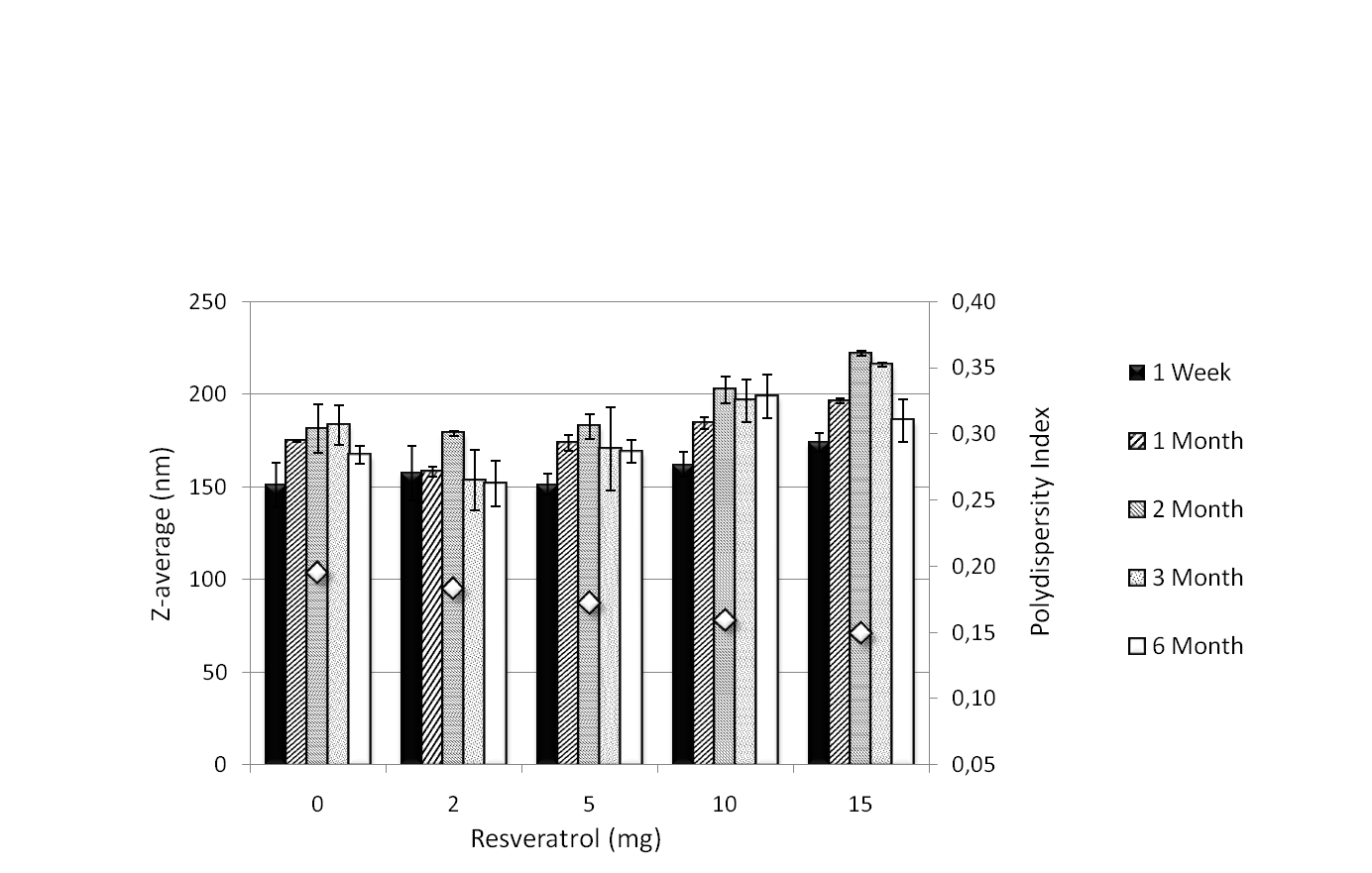

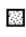

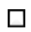


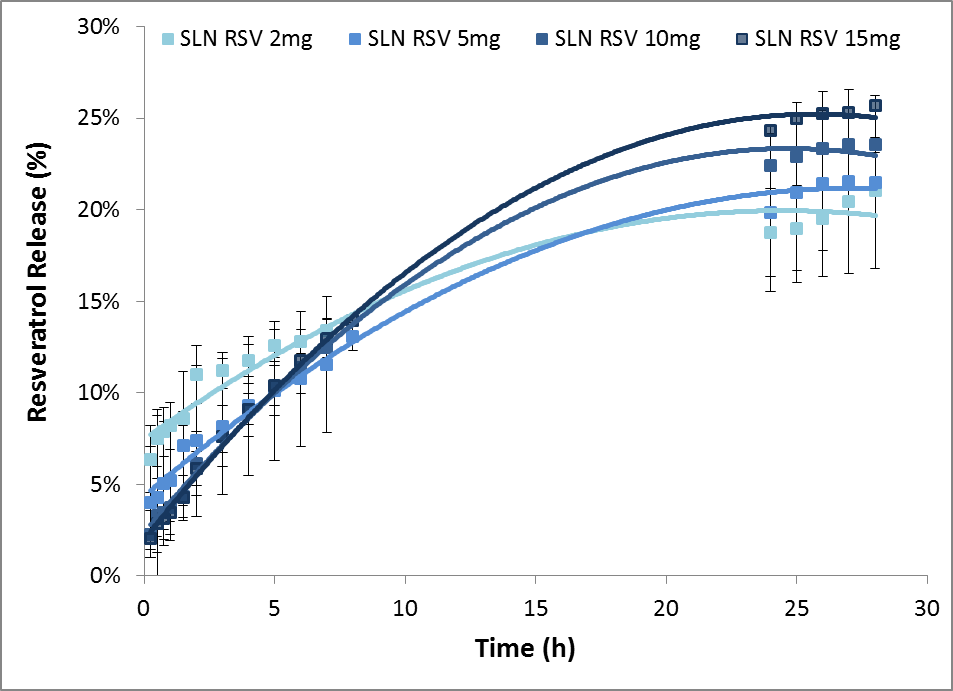


**Figure S4 –** *In vitro* RSV release profiles from RSV loaded SLNs with different amounts of RSV performed in SBF, simulating the blood stream conditions, at body temperature (37°C). All data represent the mean ± SD (n=3). No statistically significant differences have been found (P > 0.05).


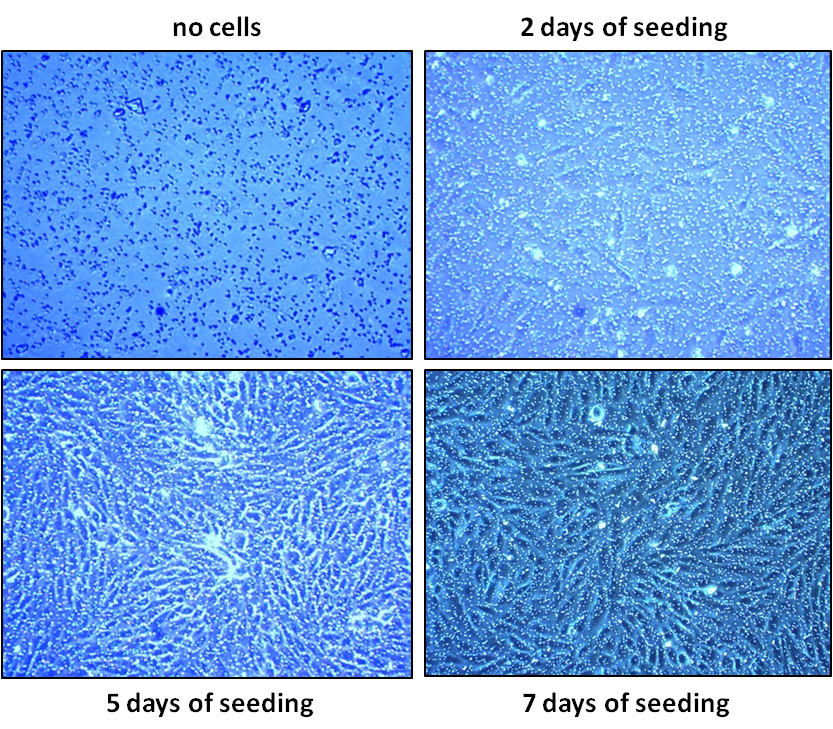


**Figure S5 –** Images of hCMEC/D3 cells on transwell devices during the 7 days of growing. Magnification 400x.
